# Experimental study of transcranial pulsed current stimulation on relieving athlete’s mental fatigue

**DOI:** 10.3389/fpsyg.2022.957582

**Published:** 2022-10-25

**Authors:** Yangyang Shen, Jian Liu, Xinming Zhang, Qingchang Wu, Hu Lou

**Affiliations:** School of Sports Science, Nantong University, Nantong, China

**Keywords:** tPCS, fatigue, non-invasive simulation on brain, fNIRS, mental fatigue

## Abstract

**Objective:**

To explore the effect of independently developed transcranial pulsed current stimulation (tPCS) on alleviating athlete’s mental fatigue.

**Methods:**

A total of 60 college athletes were randomly divided into the active stimulation group (current intensity:1.5 mA, lasting for 15 min) and the sham stimulation group. Subjective questionnaires, behavior test, and functional near-infrared spectroscopy (fNIRS) test were conducted before and after the experiment. Two-way ANOVA with repeated measures was used to compare the differences in mental fatigue indexes before and after the two experimental conditions.

**Results:**

After 7 days of exercise training, there was a significant difference in the main effect of the time factor in all indexes of the two groups (*p* < 0.05). The scores of rated perceived exertion (RPE) scale, positive and negative affect schedule (PANAS), critical flicker frequency (CFF), and reaction time (RT), in the tPCS treatment group, were better than those in the sham stimulation group (*p* < 0.05). After 7 days of exercise training, all the subjects had different degrees of athlete’s mental fatigue; the subjects in the active stimulation group have a good evaluation of the tPCS developed by the research group without adverse actions.

**Conclusion:**

tPCS intervention can improve emotional state, reduce the subjective evaluation of fatigue, improve behavioral levels such as attention and reaction time and increase cerebral prefrontal blood flow and oxygen supply.

## Introduction

Fatigue is a real problem faced by all athletes. In the field of sports, such as training and competition, when the stress condition of high energy cost and low perceived benefit of the body lasts, consumption does not match the activation level of the homeostasis control system, and the structure that is consciously perceived by the brain is mental fatigue, which is called exercise-induced mental fatigue (St Clair Gibson et al., 2003). And this phenomenon is usually accompanied by physical performance, and emotional or cognitive decline (Boksem and Tops, 2008). Recovery from mental fatigue in athletes can affect mood, cognition, and motivation, which in turn determines the rate of recovery from fatigue and training effects (Noakes, 2012; Li et al., 2019). Therefore, how to accelerate fatigue recovery is not only an important link to improving training quality, but also a hot issue in the field of sports training research.

During sustained efforts, fatigue-related performance declines over time, also known as fatigue, and can be quantified by objective measures. These include decreased processing speed, reaction time (RT), or accuracy during continuous cognitive testing (Harrison et al., 2017). In the measurement method of athlete’s mental fatigue, the Rated Perceived Exertion (RPE) scale developed by Borg (1998) is commonly used to assess the subjects’ self-perception of effort or fatigue. Some studies have required that subjects measure RPE every 20 s during the completion of the experimental task. And the outcomes showed that RPE was significantly elevated when subjects suffered from mental fatigue (Pageaux et al., 2013). Li (2020) also pointed out in his research that the RPE scale could be adopted to examine mental fatigue. Therefore, this study intended to measure mental fatigue by virtue of RPE. In addition, emotion and training state are also common subjective evaluation indicators of mental fatigue (Kellmann and Kallus, 2001; St Clair Gibson et al., 2003).

In recent years, monitoring the oxygenation changes of the cerebral cortex, especially the prefrontal cortex (PFC) through functional near-infrared spectroscopy (fNIRS) is a new approach to study mental fatigue (Zhang et al., 2017). The study found that fNIRS measurement is sensitive to mental load and practice level, and can monitor the hemodynamic changes related to mental load through fNIRS (Ayaz et al., 2012). Some researchers measured the oxygenated hemoglobin (HbO_2_) characteristics of PFC after inducing mental fatigue in subjects and found that the cerebral hemodynamics measured by fNIRS could serve as an indicator to detect mental fatigue (Zhang et al., 2017). PFC is a piece of cortex located in the frontal part of the human hemisphere and is most associated with highly developed intelligence, mental processes, and cognitive function in primates. It’s proved that mental fatigue not only has a significant effect on the correlation of prefrontal symmetric electroencephalogram (EEG) signals but also reduces the synchrony of prefrontal symmetrical-lead EEG signals (Ma, 2010; Zhang, 2020). Moreover, the contents of HbO_2_ and deoxygenated hemoglobin (HHb) are closely related to the degree of mental fatigue (Rupp and Perrey, 2008). Therefore, fNIRS was chosen to detect cerebral hemodynamics in the PFC of the brain and then to measure mental fatigue in this study.

The exact mechanisms that produce mental fatigue are not yet known, but some studies have shown that an athlete’s mental fatigue is a systematic response made by the brain after the evaluation of exercise (Swart et al., 2012). Therefore, brain stimulation may improve the level of an athlete’s mental fatigue. In recent years, non-invasive brain stimulation technology has shown great potential in alleviating athletes’ fatigue (Angius et al., 2018; Wang and Liu, 2018). It can regulate the excitability of the cerebral cortex. It is possible to alleviate an athlete’s mental fatigue by improving exercise motivation, reducing muscle pain, stimulating fatigue at the spinal cord level, and decreasing or reducing self-perceived fatigue (Yin and Liu, 2019). At present, there are abundant research results related to transcranial direct current stimulation (tDCS) (Qiao et al., 2020). Its products come from European and American countries. It is a non-invasive neural regulation technology that uses constant and low-intensity direct currents to regulate the activity of cortical neurons (Reardon, 2016; Hornyak, 2017). In some research, tDCS was performed on the prefrontal cortex of subjects after induction of cognitive fatigue, and the results showed that tDCS could reduce fatigue and had the potential to treat fatigue in neuropsychiatric disorders (Linnhoff et al., 2021). Transcranial pulsed current stimulation (tPCS) is a non-invasive neural regulation technology that selectively activates the activities of endorphins and cortical neurons with variable frequency and intensity (Lebedev et al., 2002). Because it can stimulate the deep brain and change the secretion of endorphins, it may have a good alleviating effect on athlete’s mental fatigue. Thus, it has greater research and development value than tDCS similar products.

In recent years, most studies investigated the effects of tPCS on brain function have focused on basic research, and few studies have focused on practical applications (Datta et al., 2013; Vasquez et al., 2016). Fudin et al. (2019) combined tPCS with 5-hydroxytryptamine laser penetration to intervene in athletes, and the results confirmed the effectiveness of tPCS in relieving fatigue in athletes in terms of blood parameters, behavioral parameters, and subjective questionnaires. Wu et al. (2022) showed that stimulation of the frontal lobe by tPCS may lead to top-down regulation, ultimately affecting the regulation of the hypothalamic-pituitary-adrenal (HPA) axis, inhibiting cortisol secretion, avoiding loss of muscle strength and decline in individual function, thereby maintaining motor function and delaying the accumulation of fatigue. Therefore, in order to break through the technical barriers blocked by European and American countries, this part is also included in the key special project of China’s national key research and development plan “science and technology Winter Olympics.” Based on the above study, we speculate that tPCS can effectively relieve mental fatigue in athletes and the purpose of this study is to explore the effect of tPCS on mental fatigue in atheletes.

## Materials and methods

### Participants

Sixty healthy college athletes volunteered to participate in the experiment with compensation and were randomly divided into the active stimulation group (*n* = 30) and the sham stimulation group (*n* = 30). All subjects signed the informed consent before the experiment and received certain remuneration after the experiment. This study was approved by the ethics committee of Nantong University (2020-1).

### Equipment and devices

#### Transcranial pulsed current stimulator

Transcranial pulsed current stimulation developed by the project team was used. One electrode piece was placed in the middle of the forehead, with an area of 5 cm × 9 cm. Two electrode pieces were placed at the mastoid process of the left and right ears, with two areas of 5 cm × 5 cm electrode pieces (Lebedev et al., 2001; Lebedev et al., 2002), a total of three electrodes, pulsed frequency of 60–80 Hz, current intensity adjustment ranged 0–2 mA, maximum voltage 20 V. Besides, the pulse was a bidirectional pulse with a square-wave waveform, the duty cycle was 29.7%. The instrument was composed of a switch, adjusting key, current intensity panel, output line, and power line, and the scale of the current intensity panel was 0.1 mA. According to the results of relevant studies, a current intensity of 1.5 mA was applied (Jaberzadeh et al., 2015; Khayyer et al., 2018; Yang et al., 2021). The instrument has passed the whole project inspection (Report No. chtsm21040050-52) of Shenzhen Hua Tong Wei International Inspection Co., Ltd. (with CMA, ILAC MRA, and CNAs quality inspection qualifications) and obtained the safety certification.

#### Functional near-infrared spectroscopy

Octamon+ portable wireless near-infrared brain imaging system produced by Artinis company in the Netherlands was used to monitor the prefrontal blood oxygen signal. The optical pole of Octamon+ includes eight dual wavelength light sources (760 and 850 nm) and two detectors. Eight channels are formed at a spacing of 3.0 cm, covering the PFC. The sample sampling rate is 50 Hz. The same concentration changes of frontal HbO_2_, HHb, total hemoglobin (HbTot), and hemoglobin difference (HbDiff) were obtained according to the modified Lambert–Beer law (Δμmol cm). Among them, HBO_2_ is a sensitive indicator of local cerebral blood flow changes, HHb is the change of deoxygenation state, HbTot reflects the change of local blood volume, and HbDiff is a good indicator of oxygenation when HbTot is relatively stable over time (Rupp and Perrey, 2008).

**FIGURE 1:**
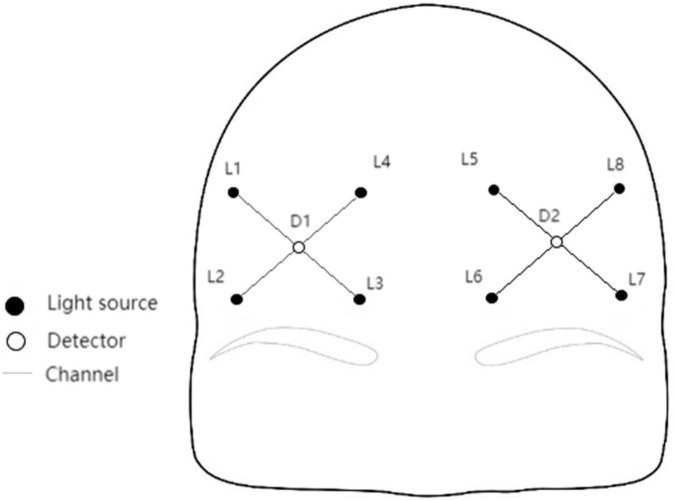
Channel layout.

After measurement of fNIRS, positions of fNIRS channels were registered to MNI spatial coordinates using a 3D localizer (FASTRAK, Polhemus, Colchester, VT, USA) and probabilistic registration methods, then fNIRS channels were determined to be located in the dorsal prefrontal cortex and the ventral prefrontal cortex. Notably, according to the international 10–20 system, L3 and L6 were placed at Fp2 and Fp1, respectively (Figure 1).

#### Behavior test

The RT tester (EP202.203) produced by Shanghai East China Normal University Science and Education Instrument Co., Ltd. was applied to compare the choice RT of the subjects under light stimulation of four colors (red, green, yellow, and blue). By the way, a four-hole photoelectric contactless response key acted as the response component of the subjects, with 20 stimuli per test (5 times for each color). At the beginning of the test, the instrument automatically and randomly presented the four-color light stimulus. According to the presentation of the light stimulus, the fingers of the subjects left the middle position of the response keyboard and pressed the circular hole of the corresponding color. The instrument automatically recorded the time between stimulus presentation and the fingers of subjects entering the circular hole of the corresponding response keyboard, and the mismatched color that subjects pressed would automatically be processed as an error by the instrument, and the corresponding time was not counted in the statistics. After completion of 20 tests, the buzzer in the instrument automatically sounded for 1 s for a hint. Finally, the total average RT was recorded (Huang and Ma, 2010).

A bright spot scintillator (EP403) produced by Shanghai East China Normal University Science and Education Instrument Co., Ltd. was applied in this study. Briefly, the flicker frequency ranged from 8.0 to 60.0 Hz, continuously adjustable. The color of the bright spot was set as red, the background light was white, the brightness/black ratio was 3:1, and the light intensity of the bright spot was 1/8. Besides, the subjects were tested alone, and the testing was performed with the forms of “↓” and “↑”. For one thing, “↑” indicated the frequency from high to low. Firstly, the experimenters turned the frequency down until the white bright spot was flashing, then the subjects were required to click the frequency increase button until the flicker disappeared, and the final frequency was recorded by the experimenters. For another, “↓” meant the frequency from high to low. The experimenters first raised the frequency of the disappearance of the bright spot flashes. Next, the subjects were asked to press the frequency reduction button until the flicker was visible, then the experimenters recorded the final frequency. Each test was performed in the order of “↓↑↑↓”, a total of four data were checked, and the arithmetic average was recorded (Zhang et al., 2020).

An attention concentration tester (EP701C) produced by Shanghai East China Normal University Science and Education Instrument Co., Ltd. was adopted for the examination of the attention concentration ability of individuals. To be specific, the instrument was placed on a table about 1.30 m high, then the subjects held the induction handle with a handedness to trace the white sign of rotation in the hexagonal track. Before measurement, the instrument was adjusted in advance, the movement speed of the white mark was set as 30 r/min, and the test time as 20s. The instrument automatically recorded the on-target time and off-target time after completion of the test. The test rules were explained first, then the subjects were allowed to practice once and measure once (Peng et al., 2017).

#### Heart rate monitoring

The first beat heart rate sensor (Firstbeat technologies, Finland) was used to record the heart rate of subjects during the whole training process. After collecting the data, the data were exported and analyzed by the first beat sports system (version 4.4.0.2, Firstbeat technologies, Finland).

#### Subjective scale

The subjective evaluation of athlete’s mental fatigue adopts the RPE10 scale. The higher the RPE score, the higher the conscious exercise intensity (Borg, 1990; Table 1).

**TABLE 1:** Borg’s CR-10 scale.

Score	Description	
0	Nothing at all	
0.5	Extremely weak	(Just noticeable)
1	Very weak	
2	Weak	(Light)
3	Moderate	
4		
5	Strong	(Heavy)
6		
7	Very strong	
8		
9		
10	Extremely strong	(Almost max)

The mental fatigue subscale in RESTQ76-Sport revised by Yan Ning (Yan, 2003) has 5 items in total, each item includes 7 scoring grades (0–6 points), and higher scores indicate a higher probability of corresponding mental situations emerging, Cronbach’s α = 0.73.

The positive and negative affect schedule (PANAS) revised by Qiu et al. (2008) includes two dimensions of positive emotion and negative emotion, with a total of 18 items; the 5-point Likert scale was applied to score, and the emotional experience intensity ranged from “very mild” to “very strong.” The positive emotion Cronbach’s α = 0.761 and the negative emotion Cronbach’s α = 0.761.

### Experimental protocol

**FIGURE 2:**
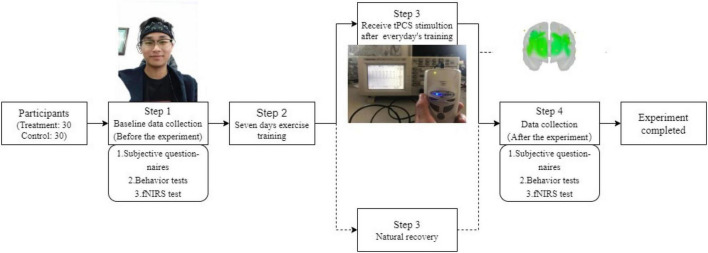
Flow diagram of experiment.

Approximately 2 (stimulation condition) × 2 (time) two factors mixed design was used in this experiment. The independent variables are group and time. The group variables are divided into two levels, that is, the tPCS active stimulation group with active stimulation and the sham stimulation group with sham stimulation. The time variables are also divided into two levels, that is, before intervention and after the intervention. The dependent variables were subjective reports, behavioral tests, and fNIRS-related indicators (Figure 2).

### Experimental procedure

Before the experiment, the subjects were randomly divided into active stimulation groups and sham stimulation group. Each subject received a subjective evaluation, behavior test, and fNIRS test from the morning before and after the whole experiment. The active stimulation group received a 15 min intervention immediately after completing exercise training every day altogether for 7 days. During the training, the coach requires each athlete to reach the degree of exhaustion (HR > 140 b/min, training duration 3 h/day) (Wu et al., 2011; Dong and Gao, 2020). The daily training contents are arranged as follows: the first day of high load physical training, mainly 800 and 1,500 m of track and field; The next day, technical and tactical training will be carried out, focusing on special technology; On the third day, increase the amount of physical training; On the fourth day, continue technical and tactical training; Maintain the amount of physical training on the fifth day; On the sixth day, conduct tactical integration training; On the seventh day, maintain the amount of physical training.

### Intervention program

This experiment is a randomized double-blind experiment. tPCS is managed by personnel who can operate and do not participate in this study. The subjects do not know the type of stimulation. The stimulation intensity and regulation process refer to Marceglia et al. (2016) and Cespón Cespon et al. (2019). At the beginning of the active stimulation condition, the operator gradually increased the current from 0 to 1.5 mA within 30 s for 15 min. At the end of the stimulation, the current automatically decreased to 0 mA within 30 s. In order to avoid the placebo effect, the sham stimulation group received sham stimulation. The sham stimulation condition used the same simulation time. At the start of the sham stimulation condition, the current was increased gradually from 0 to 1.5 mA followed by a decrease from 1.5 to 0 mA by operators within 30 s; the sham stimulation lasted for 15 min (Xiao et al., 2020). As for the active stimulation, after the initial current acceleration process, the operator readjusted the current to 0 mA. During the whole stimulation process, when the subject feels any uncomfortable feeling caused by the stimulation, stop the stimulation immediately. After each stimulation, if the subject has any discomfort, it shall be fed back to the experimenter in time and recorded.

### Statistical analysis

All data were collected by SPSS24.0 statistical software package for statistics and analysis. The measurement data are expressed as mean ± standard deviation (SD). Repeated measurement analysis of variance was used to analyze the subjective report, behavior test, and fNIRS of the two groups. Mauchly’s Test of Sphericity was performed first for determination. When the spherical hypothesis was true, the bivariate analysis of variance could be directly used for checking the result (Sphericity Assumed); when the spherical hypothesis was wrong, the bivariate analysis of variance could be adopted to correct the results (Greenhouse–Geisser for correction). Additionally, a simple effect analysis was performed when the interaction effect between the time and intervention modality was significant. Pairwise comparisons were performed using Bonferroni correction (a multiple-comparison correction applied when several independent or dependent statistical tests were conducted at the same time). The significance level was set at *p* < 0.05.

## Results

### Baseline data

The day before the experiment, the baseline data of 60 subjects were collected. There were 30 in the active stimulation group (21 males and 9 females; the average age was 20.47 ± 0.72 years, ranging from 19 to 22 years; the average training period was 4.12 ± 0.99 years, ranging from 3 to 5 years), There were 30 in the sham stimulation group (21 males and 9 females; the average age was 20.63 ± 1.46 years, ranging from 19 to 24 years; the average training period was 4.00 ± 0.75 years, ranging from 3 to 5 years). The test results revealed that there were no significant differences in the parameters of baseline data between the two groups (*p* > 0.05) and the two groups were homogeneous (Tables 2–4).

**TABLE 2:** Baseline data analysis of subjective report test of two groups of subjects.

	PANAS scale		RPE	RESTQ76-Sport
	PA	NA		
Active stimulation group	31.23 ± 7.42	11.53 ± 3.15	4.07 ± 2.33	7.17 ± 4.34
Sham stimulation group	31.60 ± 6.39	12.07 ± 1.87	3.83 ± 2.18	7.00 ± 5.56
*t*	−0.21	−0.80	0.40	0.13
*p*	0.84	0.43	0.69	0.90

PANAS, positive and negative affect schedule; PA, positive affect; NA, negative affect; RPE, rate of perceived exertion; RESTQ76-Sport, the Recovery-Stress Questionnaire for Athletes.

**TABLE 3:** Analysis of baseline data of behavior test of two groups of subjects.

	CFF (Hz)	RT (s)	attention	
			Duration target (s)	Miss times
Active stimulation group	40.00 ± 2.85	0.37 ± 0.10	14.14 ± 1.64	42.30 ± 5.69
Sham stimulation group	40.88 ± 2.83	0.37 ± 0.13	14.01 ± 1.46	41.17 ± 6.10
*t*	−1.20	−0.23	0.32	0.74
*p*	0.24	0.82	0.75	0.46

CFF, critical flicker frequency; RT, reaction time.

**TABLE 4:** Analysis of baseline data of oxygenated hemoglobin in two groups.

	HbO_2_	HHb	HbTot	HbDiff
Active stimulation group	−6.88 ± 8.19	4.06 ± 2.73	12.77 ± 9.74	3.37 ± 5.19
Sham stimulation group	−7.50 ± 7.23	4.46 ± 2.24	12.91 ± 8.34	3.62 ± 5.01
*t*	0.31	−0.62	−0.06	−0.19
*p*	0.76	0.54	0.95	0.85

HbO_2_, oxyhemoglobin; HHb, deoxyhemoglobin; HbTot, total hemoglobin; HbDiff, hemoglobin difference.

### User evaluation of transcranial pulsed current stimulation

The following four items were set in the Subjective Perception of Experimental Procedures Questionnaire. Answers were coded on a 7-level Likert scale (1 = strongly disagree, 7 = strongly agree) with Cronbach’s α = 0.711. And the outcomes revealed that subjects did not suffer any adverse reactions during the experiment and none of the scores for the four items in the questionnaire exceeded 5 points, indicating that transcranial pulsed current stimulator had good comments among users receiving athlete’s mental fatigue intervention. It’s not ignored that there was a significant difference between the two groups. Specifically, the subjects in the sham stimulation group did not receive tPCS stimulation, so they did not feel the tremor caused by the pulse stimulation and had a much lower discomfort score during the experiment compared with the active stimulation group (Table 5).

**TABLE 5:** Subjective Perception of Experimental Procedures Questionnaire.

Items	Q1	Q2	Q3	Q4
Active stimulation group	1.83 ± 0.60	1.73 ± 0.52	1.63 ± 0.50	1.90 ± 0.40
Sham stimulation group	1.40 ± 0.56	1.27 ± 0.45	1.13 ± 0.35	1.40 ± 0.62
T	2.90	3.71	4.57	3.70
*p*	<0.01**	<0.01**	<0.01**	<0.01**

Q1, I reject the tremors and numbness caused by electrical stimulation; Q2, I feel very uncomfortable in the stimulation intervention; Q3, I feel very painful during stimulation intervention; Q4, I am not willing to receive pulse stimulation any more. **Indicate *p* < 0.01, the statistics were significantly different.

### Effect of transcranial pulsed current stimulation on a subjective scale of athlete’s mental fatigue

**FIGURE 3:**
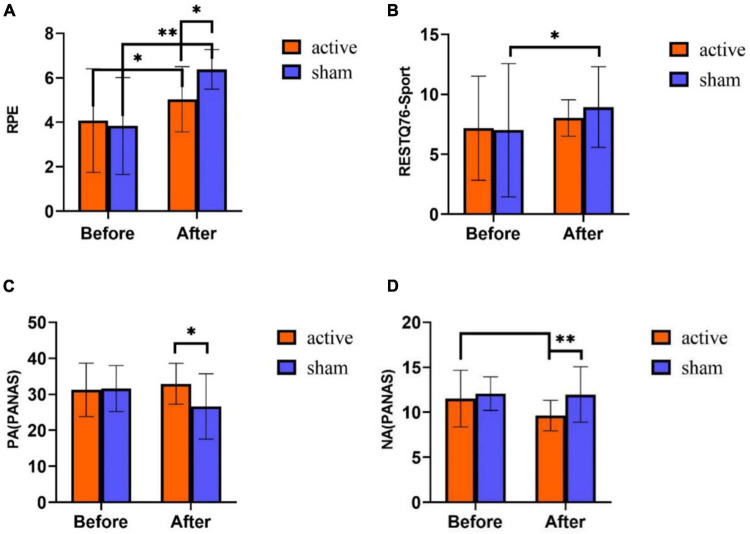
Subjective report results of tPCS on athletic mental fatigue. **(A)** Rated perceived exertion (RPE) scale; **(B)** RESTQ76-Sport Questionnaire; **(C)** positive affect schedule (PANAS); **(D)** negative affect schedule (PANAS). *p < 0.05, ^**^p < 0.01.

On RPE score, the results showed that the main effect of time was significant [*F* (1,58) = 48.348, *p* < 0.01], the main effect of intervention mode was not significant [*F* (1,58) = 1.937, *p* = 0.169], and the interaction between time and intervention mode was significant [*F* (1,58) = 9.687, *p* = 0.003]. *Post-hoc* test analyses (Figure 3A) revealed that there was no significant difference in RPE scores between the two groups before training (*p* = 0.691). The RPE scores of both active stimulation and sham stimulation groups were significantly higher after training than before training, but the RPE scores of the active stimulation group were significantly lower than those of the sham stimulation group (*p* < 0.05).

On RESTQ-76 Sport score, the main effect of time was significant [*F* (1,58) = 4.888, *p* = 0.031], the main effect of intervention mode was not significant [*F* (1,58) = 0.272, *p* = 0.604], and the interaction effect of time and intervention mode was not significant [*F* (1,58) = 0.709, *p* = 0.403]. Statistical analysis of the time main effects revealed significantly higher RESTQ-76Sport scores on the post-test of the experiment than on the pre-test (*p* < 0.05) (Figure 3B).

In the PA score of PANAS scale, the main effect of time was not significant [*F* (1,58) = 3.229, *p* = 0.078], the main effect of intervention mode was not significant [*F* (1,58) = 3.289, *p* = 0.075], and the interaction effect of time and intervention mode was significant [*F* (1,58) = 13.448, *p* < 0.001). *Post hoc* test analyses (Figure 3C) exhibited that there was no significant difference in PA scores between the two groups before the training (*p* = 0.838). Nevertheless, after training, the scores in the active stimulation group were significantly higher than those in the sham stimulation group (*p* = 0.002). Before and after training, PA scores had no obvious differences in the active stimulation group (*p* = 0.210), while the sham stimulation group exhibited a significant difference in PA scores before and after training (*p* < 0.001).

On the NA score of PANAS scale, the main effect of time was significant [*F* (1,58) = 6.266, *p* = 0.015], the main effect of intervention mode was significant [*F* (1,58) = 7.613, *p* = 0.008], and the interaction effect of time and intervention mode was significant [*F* (1,58) = 5.075, *p* = 0.028]. The results of Figure 3D showed no significant difference in NA scores between the two groups before training (*p* = 0.53). After training, the scores in the active stimulation group were significantly lower than the sham stimulation group (*p* = 0.001). In the active stimulation group, the scores after training were obviously lower than those before training (*p* = 0.001). In the sham stimulation group, NA scores had no significant difference before and after training (*p* = 0.133).

### Effect of transcranial pulsed current stimulation on athlete’s mental fatigue behavior test

**FIGURE 4:**
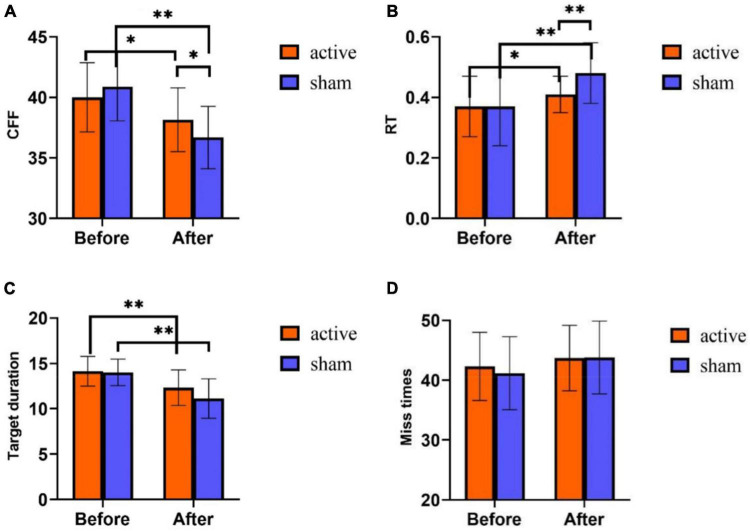
Behavior test results of tPCS on athletic mental fatigue. **(A)** Critical flicker frequency (CFF); **(B)** reaction time (RT); **(C)** target duration (attention); **(D)** miss times. *p < 0.05, ^**^p < 0.01.

Repeated measurement ANOVA was used to analyze the differences in time and intervention mode in the three indicators of the behavior test. The results showed that in CFF, the main effect of time was significant [*F* (1,58) = 43.552, *p* < 0.01], the main effect of intervention mode was not significant [*F* (1,58) = 0.304, *p* = 0.584], and there was an interaction between time and intervention mode [*F* (1,58) = 6.534, *p* = 0.013]. *Post-hoc* test analyses (Figure 4A) disclosed that both groups had no significant difference in CFF before training (*p* = 0.235). After training, CFF was significantly higher in the active stimulation group than in the sham stimulation group (*p* = 0.033). Furthermore, CFF after training was significantly lower than that before training both in the active stimulation group and the sham stimulation group (*p* < 0.001).

On RT, the main effect of time was significant [*F*(1,58) = 25.808, *p* < 0.001], the main effect of intervention mode was not significant [*F* (1,58) = 3.583, *p* = 0.063], and there was an interaction between time and intervention mode [*F* (1,58) = 5.064, *p* = 0.028]. *Post-hoc* test analyses (Figure 4B) exhibited no differences in RT between the subjects of the two groups before training (*p* = 0.819). While after training, RT showed a huge difference between the two groups (*p* = 0.001), and RT was much lower in the active stimulation group than in the sham stimulation group. Additionally, both in the active and sham stimulation groups, RT was much higher after training than before training (*p* < 0.05).

On the target duration (attention), the main effect of time was significant [*F* (1,58) = 45.364, *p* < 0.001], the main effect of intervention mode was significant [*F* (1,58) = 4.362, *p* = 0.041], and the interaction effect of time and intervention mode was not significant [*F* (1,58) = 2.394, *p* = 0.127]. Statistical analysis of the time main effect (Figure 4C) revealed that the target duration was significantly lower after training than before training.

In the number of missed targets (attention) (Figure 4D), the main effect of time was not significant [*F* (1,58) = 3.565, *p* = 0.064], the main effect of intervention mode was not significant [*F* (1,58) = 0.235, *p* = 0.630], and the interaction effect of time and intervention mode was not significant [*F* (1,58) = 0.333, *p* = 0.566].

### Effect of transcranial pulsed current stimulation on athlete’s mental fatigue functional near-infrared spectroscopy

**FIGURE 5:**
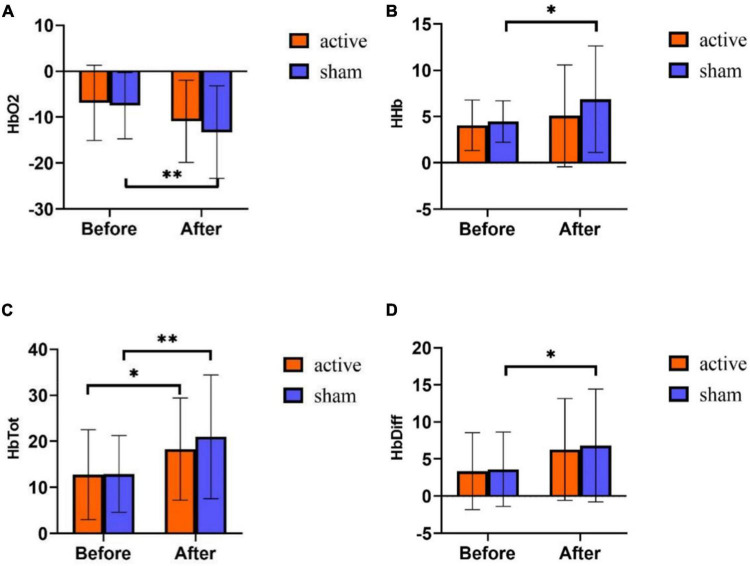
fNIRS results of tPCS on athletic mental fatigue. **(A)** Oxyhemoglobin (HbO_2_); **(B)** deoxyhemoglobin (HHb); **(C)** total hemoglobin (HbTot); **(D)** hemoglobin difference (HbDiff). *p < 0.05, ^**^p < 0.01.

Repeated measurement ANOVA was used to analyze the differences in time and intervention mode in the four indexes of near-infrared. The results showed that on HbO_2_, the main effect of time was significant [*F* (1,58) = 10.942, *p* = 0.002], the main effect of intervention mode was not significant [*F* (1,58) = 0.795, *p* = 0.376], and the interaction effect of time and intervention mode was not significant [*F* (1,58) = 0.356, *p* = 0.553]. Statistical analysis of the main effect of time (Figure 5A) showed that the content of HbO_2_ was significantly lower after training than before training.

On HHb, the main effect of time was significant [*F* (1,58) = 4.892, *p* = 0.031], the main effect of intervention mode was not significant [*F* (1,58) = 1.809, *p* = 0.184], and the interaction effect of time and intervention mode was not significant [*F* (1,58) = 0.823, *p* = 0.368]. Statistical analysis of the time main effect (Figure 5B) revealed that HHb was significantly higher after exercise training than before training.

On HbTot, the main effect of time was significant [*F* (1,58) = 14.537, *p* < 0.001], the main effect of intervention mode was not significant [*F* (1,58) = 0.430, *p* = 0.514], and the interaction effect of time and intervention mode was not significant [*F* (1,58) = 0.510, *p* = 0.478]. Statistical analysis of the time main effect (Figure 5C) revealed that HbTot was significantly higher after training than before training.

On HbDiff, the main effect of time was significant [*F* (1,58) = 8.879, *p* = 0.004], the main effect of intervention mode was not significant [*F* (1,58) = 0.097, *p* = 0.757], and the interaction effect of time and intervention mode was not significant [*F* (1,58) = 0.020, *p* = 0.889]. Statistical analysis of the time main effect (Figure 5D) showed that HbDiff was significantly higher on the post-test of the experiment.

## Discussion

This study aims to alleviate athletes’ mental fatigue, and uses tPCS independently developed by the research group as an intervention means to explore the impact of tPCS on subjective reporting, behavior test, and fNIRS of athletes’ mental fatigue. The results are consistent with the hypothesis. After tPCS intervention for seven consecutive days, the active stimulation group effectively alleviated the athlete’s mental fatigue. Specifically, the time main effects of the subjective report, behavior test, and fNIRS were significant, indicating that 7-day exercise training caused mental fatigue in the two groups of subjects, but the indexes of athlete’s mental fatigue in the active stimulation group stimulated by tPCS were significantly better than those in the sham stimulation group. These results show that tPCS intervention for seven consecutive days can play a positive role in alleviating an athlete’s mental fatigue. In this study, after the tPCS layout scheme was set as PFC and binaural, the subjects did not report discomfort and the intervention effect was good, which was different from the main layout in the motor area in the tDCS-related studies. We believe that an athlete’s mental fatigue can be regulated by a variety of mental factors, such as emotion, motivation, and will, and PFC may play a role in integrating this information to alleviate fatigue (Miller and Cohen, 2001). In addition, previous studies have shown that the oxygenation level of PFC has decreased significantly in fatigue tasks, which is consistent with the findings of this study. And the change of PFC can reflect the redistribution of blood oxygen to more important and active parts of the brain, so as to ensure that the metabolism of relevant regions matches the degree of fatigue consumption (Jung et al., 2015). Mental fatigue in atheletes leads to the reduction of blood oxygen in relevant brain regions, and tPCS for PFC can increase the supply of blood oxygen, so as to accelerate the approach to the steady-state level and reduce the sense of fatigue. In all four indicators of fNIRS, the rate of change before and after training was lower in the active stimulation group than in the sham stimulation group. It also confirmed that the subjects were in a more stable state under the intervention effect of tPCS.

After 7 days of exercise training, the subjective feelings of fatigue of the subjects in the active and sham stimulation groups were increased at different levels, but the indexes of the active stimulation group were significantly better than those of the sham stimulation group. Among them, tPCS has an obvious alleviating effect on the RPE index, and RPE is a commonly used questionnaire to evaluate subjective feelings of fatigue (Marcora et al., 2009; Pageaux et al., 2015; Smith et al., 2015). RPE can measure perceived fatigue, also known as perceived effort, which can be defined as consciously feeling how difficult, heavy, and tired a sport task is. Current research suggests that the brain processes neural signals to generate the perception of effort, including afferent feedback from motion-related muscles and other peripheral physiological systems, as well as neural signals related to central motor instructions (Pageaux, 2016). tPCS alleviating fatigue may be due to changing the processing of neural signals as the basis of effort perception, thereby reducing the afferent feedback intensity of the peripheral physiological system and the intensity of central motor command, so as to reduce the subjective degree of perceived fatigue.

Emotion plays a key role in an athlete’s mental fatigue (Tempest and Parfitt, 2016). Our results also prove that there is no significant difference between the positive emotion of the active stimulation group before and after the intervention, indicating that tPCS can effectively maintain the positive emotion of athletes, while the positive emotion of the sham stimulation group decreased significantly; For negative emotions, the negative degree after tPCS intervention can even be lower than that before exercise training. This is different from the relevant studies of tDCS. It may be because tPCS has a regulatory effect on the content of endorphins (Lebedev et al., 2002) or has a positive effect on the structure of endorphins (Lebedev et al., 2001), which is also an important difference between tPCS and tDCS in the intervention mechanism. Endorphins are considered modulators of mood and satisfaction. Increasing the content of endorphins through intervention can show an increase in positive emotions such as happiness, and a decrease in negative emotions such as anxiety and depression (Sher, 1996). Endorphins also play a role in mental fatigue, and their increased concentration may weaken the subjective feeling of fatigue (Harber and Sutton, 1984). As a means of recovering from mental fatigue after daily sports training, tPCS can alleviate the negative emotion brought by sports training, maintain the level of positive emotion, alleviate the increase of sense of fatigue, and improve mental state.

In the present study, regarding the RESTQ-76 scale, we selected five entries of the mental fatigue dimension of the revised RESTQ-76 scale by Zeng and Yan (2007). The results showed no significant differences in RESTQ-76 scores between the active and sham stimulation groups. Cross-cultural testing of the scale by Zeng and Yan (2007) indicated that there was a crossover between the dimensions of the scale, resulting in no significant difference in effects after subjects received a single tPCS intervention in the prefrontal lobes *versus* behind both ears. This is consistent with the results of the present study.

Although the behavioral indexes of the subjects decreased after exercise training, the t PCS active stimulation group was better than the sham stimulation group in CFF, RT. With the deepening of fatigue, CFF changes from fusion flash to continuous intermittent light spot, and RT gradually slows down (Qi, 2006). Behavioral indicators can reflect the excitement of reaching the visual center through the optic nerve from the orbitofrontal region. TPCS may improve the excitement level of the visual system and reduce the fatigue effect by stimulating the PFC. The active stimulation group may also improve the reaction speed due to the decrease in negative emotion. Lv (2008) also support this view. She found that vigilance and attention before and after fatigue are related to negative emotions. In addition, there was no significant difference in attention (target duration) between the two groups in the present study. Attention is not a single structure and is not associated with a single restricted brain region. It is the result of the interaction of different brain areas organized in the network (Corbetta and Shulman, 2002; Corbetta et al., 2008). This means that tPCS stimulates prefrontal and postauricular brain regions, which may not be stimulated enough to cause an increase in attentional capacity. It has also been shown that the more automatic or stimulus-driven processes in the brain are relatively unaffected by mental fatigue (Boksem et al., 2005), resulting in a non-significant time, group main effect and interaction effect for the number of times the subjects were off-target.

The change of brain oxygenation reflects the regulation of brain function activation and the degree of athlete’s mental fatigue (Bhambhani, 2012). Although the PFC may not be as directly involved in the neural control of movement as the motor area, it is located “upstream” of the motor cortex and indirectly participates in the motor control. This is consistent with the view of Taylor et al., who believe that the signal changes related to fatigue may be regulated by the “upstream” cortex of the motor cortex, rather than by the neural structure of the motor cortex itself or more “downstream” (Taylor et al., 2000). Rasmussen et al. (2007) also showed that the decrease of prefrontal cortical oxygenation was related to the occurrence of fatigue. In the present study, the fNIRS scores were all significant for the time main effect, indicating that the subjects developed mental fatigue after one week by training. In addition, the main effect of the group and the interaction effect of time and group was not significant, which was different from the results of previous studies. In fact, one week after the tPCS intervention, there was no significant difference between the two groups. However, the state of the subjects in the active stimulation group was closer to the baseline state, which is some evidence that tPCS can accelerate fatigue recovery by promoting prefrontal oxygenation activity and increasing local oxygenation. In addition, Faria et al. (2011) found that the intervention effect of tDCS was not only related to the stimulated brain area but also has a more direct relationship with the current parameters such as stimulation electrode position and stimulation current magnitude. And the current path of transcranial electrical stimulation may not specify a single route, and it is more likely to spread to various brain parts (Sasada et al., 2020). This may lead to a discrepancy between the results of this experiment and the existing studies.

The main limitations of the study are as follows: First, the tPCS parameters set in this study are based on the commonly used data of transcranial electrical stimulation (i.e., 1.5 mA and 15 min), and whether this is the best dose parameter combination, or whether there are different stimulation doses for different fatigue types and degrees needs to be further discussed in the future. Second, fNIRS only detects eight channels of signals in the region of interest, and the oxygenation of the whole brain is not clear. Therefore, more channels of fNIRS can be used in the future to investigate the impact of tPCS on other brain regions. Third, the subjects are ordinary college athletes. Their sports ability, training level, and training years are different from those of high-level athletes. It is not clear whether tPCS has the same effect. In the next step, we will carry out the intervention research of Winter Olympic athletes, optimize the personalized scheme of tPCS intervention, and improve the application effect of fatigue recovery.

## Conclusion

TPCS intervention can improve emotional state, reduce the subjective evaluation of fatigue, and improve behavioral test levels such as attention and RT. Its mechanism is related to increasing prefrontal blood flow and cerebral oxygen supply. It is suggested that the developed tPCS instrument can be used to alleviate athletes’ mental fatigue.

## Data availability statement

The datasets presented in this study can be found in online repositories. The names of the repository/repositories and accession number(s) can be found in the article/Supplementary material.

## Ethics statement

This study was approved by the Ethics Committee of Nantong University (2020-1). The individual(s) provided their written informed consent for the publication of any identifiable images or data presented in this article.

## Author contributions

YS and HL: study concept and design, drafting of the manuscript, and critical revision of the manuscript for important intellectual content. JL, XZ, and QW: analysis and interpretation of data and statistical analysis. All authors study supervision, read, and approved the manuscript.
